# Group Clinical Supervision for midwives and burnout: a cluster randomized controlled trial

**DOI:** 10.1186/s12884-022-04657-4

**Published:** 2022-04-11

**Authors:** Christine Catling, Helen Donovan, Hala Phipps, Simeon Dale, Sungwon Chang

**Affiliations:** 1 Centre for Midwifery, Child and Family Health, Faculty of Health, University of Technology Sydney, Ultimo, Australia; 2 Sydney Institute for Women, Children & their Families, Sydney Local Health District, Camperdown, Australia; 3 Nursing Research Institute, St Vincent’s Health Network Sydney, St Vincent’s Hospital Melbourne and Australian Catholic University, Sydney, Australia; 4 School of Nursing, Midwifery and Paramedicine, Australian Catholic University, Sydney, Australia; 5 Centre for Improving Palliative, Aged and Chronic Care through Clinical Research and Translation (IMPACCT), Faculty of Health, University of Technology Sydney, Ultimo, Australia

**Keywords:** Midwifery, Support, Clinical supervision, Cluster randomised controlled trial, Burnout, Workplace culture

## Abstract

**Background:**

There are major shortfalls in the midwifery workforce which has been exacerbated by the COVID 19 pandemic. Midwives have high levels of burnout and many, often early career midwives, are planning to leave the profession. There are reports of a poor workplace culture in maternity units, including bullying. Support is essential for the welfare of the workforce to be able to cope with the demands of their jobs. Supportive strategies, such as Clinical Supervision, a recognised approach in healthcare, enable reflection in a facilitated, structured way, and can enhance professional standards. The purpose of this research is to study burnout levels in midwives, those exiting their workplace and perceptions of workplace culture in relation to access to, and attendance of, monthly Clinical Supervision.

**Methods:**

This study will be a cluster randomised controlled trial of maternity sites within Sydney and the surrounding districts. Twelve sites will be recruited and half will receive monthly Clinical Supervision for up to two years. Midwives from all sites will be requested to complete 6-monthly surveys comprising validated measurement tools: the Copenhagen Burnout Inventory (CBI), the Australian Midwifery Workplace Culture (AMWoC) tool and the Clinical Supervision Evaluation Questionnaire (CSEQ) (the latter for intervention sites only). Primary outcomes are the levels of burnout in midwives (using the CBI). Secondary outcomes will be the quality of the intervention (using the CSEQ), perceptions of workplace culture (using the AMWoC tool) and midwives’ intention to stay in their role/profession, as well as sick leave rates and numbers of exiting staff. We will also determine the dose effect – ie the impact in relation to how many Clinical Supervision sessions the midwives have attended, as well as other supportive workplace strategies such as mentoring/coaching on outcomes.

**Discussion:**

Through attending monthly Clinical Supervision we hypothesise that midwives will report less burnout and more positive perceptions of workplace culture than those in the control sites. The potential implications of which are a productive workforce giving high quality care with the flow-on effect of having physically and psychologically well women and their babies.

**Trial registration:**

The ACTRN Registration number is ACTRN12621000545864p, dated 10/05/2021,

## Background

Midwives are essential to maternal wellbeing. Globally, they are pivotal to the reduction of maternal and neonatal morbidity and mortality [1, 2]. This is also the case in high-resource countries such as Australia. For instance, a Cochrane review of over 17,600 women demonstrated that services offering midwife-led continuity of care resulted in a 24% reduction in preterm birth, compared with standard hospital care [3]. Other high-level research indicates that midwife-led care is associated with improved physical and psychological outcomes for mothers [4, 5], including less epidurals, episiotomies and instrumental births. Naturally, these favourable outcomes are dependent on having a midwifery workforce.

There were nearly 50,000 more births each year in Australia in 2020 than there were in 2001 [6], meaning that midwives are busier than ever. Further, there is now more diversity and complexity around birth due to factors such as rising maternal age and higher rates of obesity. Copious documentation and computerised systems designed to streamline processes can be seen as disruptive to optimal midwifery care, and force midwives to be ‘with documentation’ instead of ‘with woman’ (the origins of the meaning of a ‘mid-wife’) [7, 8]. These factors mean more midwives are necessary in the workplace but staffing ratios have not changed to reflect this acuity, contributing to the dissatisfaction and burnout of midwives across Australia.

New South Wales, the most populous state in Australia, faces a predicted shortfall of more than 8000 registered nurses and midwives by 2030 [9]; however, nuanced data regarding midwifery attrition in the workforce is not available. This modelling was completed before the pandemic: the actual shortfall is likely to be far greater. Australia appears to be educating a sufficient number of midwives to meet the needs of maternity units [10] but reports of staff shortages causing workload stress and burnout^1^ are rife [11]. The COVID 19 crises is responsible for a significant worsening of midwifery working conditions, with international reports of depression and anxiety in midwives [12], a relationship between mental health and turnover [13] and moral distress [14]. The pandemic has compounded many issues within the midwifery workforce that were problematic prior to 2020 [8] with the recognition that burnout is higher than the nursing discipline [15].

Research from the UK found that midwives who left the profession cited dissatisfaction with staffing levels, workload and conditions [16]. Midwives emphasised that they received limited support from managers and experienced constraints on their ability to provide quality care. This is similar in Australia, where midwives often report frustrations in their efforts to practice woman-centred care due to an increasingly medicalised environment [8, 17]. The international Work, Health and Emotional Lives of Midwives (WHELM) study, which included Australia, reported that midwives were discontent with the conditions in which they were required to practice [18]. They believed that workplace structures compromised the standard of care they were able to deliver, and thereby affected the relationships they established with the women in their care. Midwives in this study also cited issues with heavy workloads, which they attributed to insufficient staffing and unsupportive managers. This led to high levels of personal and work-related distress. These findings are supported in other Australia-based research that similarly indicate that midwives are not given adequate support to provide satisfactory health care to women [11, 19–21]. This can cause moral distress for midwives and contribute to burnout [14].

Australian research has also demonstrated high levels of stress and burnout among midwives, which have a direct impact on midwives’ dissatisfaction and their stated intentions to leave the profession [22–24]. One study reported that 47% of midwives working in Western Australian hospitals intended to leave the profession, 24% of them within five years [25]. Another Australian study of 1037 midwives reported that 43% had recently considered leaving the profession [8]. Both studies cited midwives’ discontent with their roles. Previous research has also uncovered evidence of a negative culture in Australian midwifery workplaces, reflected in constraints on collaboration, hierarchical practices, and uncaring or ‘toxic’ work environments [26–28].

Importantly, some studies have shown that adequate resources, support from managers, and feelings of control and empowerment are key factors in supporting the retention of midwives [29, 30]. There is clearly a need to investigate how to support midwives in these key areas to improve satisfaction, maintain quality of care and retain them in the workforce. One possible way of doing this is through the implementation of Group Clinical Supervision (GCS). The NSW Health Education and Training Institute (HETI) endorses and recommends GCS as a means for clinicians, including midwives, to reflect on their care-giving practices and to receive support “to ultimately enhance and maintain the quality and safety of patient care” [31, 32]. HETI recommends that GCS be provided for 1–1.5 h per month to enhance midwives’ emotional wellbeing and promote working relationships in supportive environments [33]. GCS is already widely applied for mental health professionals due to their stressful work environment [34]. The work environment for midwives is similarly stressful, both physically and emotionally, because they deal with stillbirth and fetal/maternal morbidity on a regular basis, but nonetheless have little access to formalised support structures [35]. This is despite a clear position statement from the Australian College of Midwives recommending GCS be implemented by professional bodies [36], and the ongoing facilitation training of senior clinicians in NSW in GCS in recent years. It is therefore vital to encourage the implementation of GCS in maternity units to improve the above-stated experiences of midwives and, as a result, the wellbeing of childbearing women and their families.

This study is a cluster randomised controlled trial of maternity sites within Sydney and the surrounding districts. The international significance and benefits of the research project will be substantial. This will be the first longitudinal research study to examine the complex relationships between workplace culture, staff attrition, burnout and sustained GCS within midwifery. The outcomes have significant implications for individual midwives, maternity services and wider healthcare systems. Moreover, the project will contribute to the development of a sustainable and committed midwifery workforce, which will be vital for ensuring maternal and infant wellbeing and providing improvements in future maternity care practices.

The project will be of considerable value to maternity managers within health organisations, not only because of its potential to reduce staff turnover costs, but through the creation of new knowledge and the enhanced capacity to build a robust, efficient, collaborative and engaged midwifery workforce. Innovative remedies to stem the midwifery attrition rate amid predicted shortfalls will benefit the Australian community, and, in particular, health organisations, women and their families. This could be life-changing for many women at this significant yet vulnerable time of their lives.

## Methods/design

The multicentre parallel cluster randomised controlled trial (RCT) will comprise 12 maternity sites with a primary objective of determining burnout levels of midwives in relation to the provision of Group Clinical Supervision (GCS), the intervention. Secondary objectives will be measuring perceptions of workplace culture and the efficacy of the GCS intervention itself in relation to GCS. Staff attrition and sick leave rates of midwives will also be measured during the study period.

It is hypothesised that regular GCS will improve midwives’ perceptions of their workplace culture and lower their levels of work-related burnout (primary outcome); that midwives with positive perceptions of their workplace culture will be more likely to remain in their profession; and maternity units that support midwives to attend regular GCS will have lower levels of midwifery staff attrition and sick leave than those that do not (secondary outcome).

Quantitative and qualitative data will be collected in this study. Validated quantitative tools will be used: the Australian Midwifery Workplace Culture (AMWoC) instrument [37], the Copenhagen Burnout Inventory (CBI) [38] and the Clinical Supervision Evaluation Questionnaire (CSEQ) [39] (intervention sites only). These are described more fully below. The survey platform REDCap ([Research Electronic Data Capture] a secure web application for building and managing online surveys and databases) will be used.

Participants in the intervention sites will be requested to attend GCS sessions as much as is practicable, but at least twice in a six-month period. Surveys will be emailed to participants every six months that contain the tools listed above, and simple demographic/workplace questions. Participant surveys in control sites will not contain the CSEQ. The number of GCS sessions or other supportive strategies (such as mentoring/coaching) that all participants have undertaken will be measured. The CONSORT statement for cluster RCTs will be used to ensure rigour and process [40].

### Governance

Governance of the study will be undertaken through a Trial Steering Committee (TSC) comprising an experienced Trial Specialist, biostatistician, midwife, a Principal Investigator (of one of the maternity sites), research assistant, Chief Investigator and an independent^2^ Chair. The role of the Steering Committee is to provide overall supervision of the study, to monitor and supervise the progress of the study towards achieving its objectives. The Committee will concentrate on the progress of the study, adherence to the protocol, safety of the midwives and consideration of new information relevant to the research question. They will also monitor recruitment rates, develop strategies to deal with any recruitment issues and approve any amendments that need further ethical authorisation.

Members of the TSC will be offered authorship of all publications, of which there are no restrictions. A publication plan has been disseminated to the TSC and the Principal Investigators with a timeframe and explanation of what authorship means. All results will be published regardless of the magnitude or direction of effect. This will involve journal publications and conference presentations, as well as reports to the Local Health Districts and/or Ministry of Health, NSW. No professional medical writers will be employed.

### Setting

The study will recruit twelve [12] predominantly Sydney-based (Australia) public maternity sites to the cluster RCT (not private facilities). Within these sites, we will recruit around 75 midwives per site (*n* = 894), a reasonable number given the numbers of midwives employed at the participating institutions. The maternity sites are Sydney-based for logistical reasons as the GCS facilitators plan to deliver the monthly Group Clinical Supervision sessions face-to-face.

### Recruitment process

Potential participants will comprise all Registered Midwives (except those in management/senior roles or agency midwives) from the participating maternity sites within Sydney and surrounds. Engagement with the Directors of Nursing and Midwifery and the Midwifery Unit Managers of antenatal, postnatal and intranatal areas of the maternity units will be undertaken, with named Principal Investigators (PIs) at each site. The PIs will contact the eligible midwives via email and through staff meetings to inform them about the study. The Midwifery Unit Managers will be reminded by the PIs of the sessions each month – so that they may release the staff from workplaces/arrange cover in order for them to attend the GCS sessions.

Multiple sessions will be held at the maternity sites to discuss the study with midwives. This will contain information about GCS and its potential benefits to the participants, and the completion of six-monthly surveys. Flyers for the study will be placed in maternity site tearooms and other high traffic areas for midwives in the maternity units. The dates and times of the GCS sessions will be co-created with each maternity site, and these may change over the course of the study to help midwives from different maternity areas participate and attend the GCS sessions. Close attention will be paid to this part of the study to ensure the highest level of participation.

Following consent, ethical approval, randomisation and extensive site liaison, the PIs at the intervention sites will alert eligible participants (midwives) and encourage attendance at the study GCS sessions on-site every month. All participants will be sent an online survey to complete at the beginning of the study and every six months during the study data collection period (planned for May 2022 – November 2024). Study information and consent for participants to complete the survey will be embedded into the online platform prior to commencement. Hardcopies of the survey will also be available for completion by participants.

### Participant information and consent

The study will be conducted in compliance with all stipulations of this protocol, the conditions of ethics committee approval, the NHMRC National Statement on Ethical Conduct in Human Research [41] and the Note for Guidance on Good Clinical Practice (CPMP/ICH-E6(R2). Participants will be provided with complete information regarding the study through the Participation Information Statement, via the Principal Investigator for the site (senior Registered Midwives), the research assistant or Chief Investigator. They will be given information about the Group Clinical Supervision (GCS) intervention and the voluntary nature of attending sessions and completing the 6-monthly surveys. Distress protocols have been formulated for use by the GCS facilitators, and Employee Assistance Program telephone numbers (for free counselling) will be embedded into the online survey and stated on the hardcopy version. Should any harms to participants occur, these will be brought to the attention of the TSC for discussion, guidance and reporting. Given the nature of the Group Clinical Supervision intervention, there are few harms anticipated for participants. Should participants feel they do not benefit from the sessions, they simply will not attend. Harms for the Group Clinical Supervision facilitators will be mitigated through monthly sessions with their own Clinical Supervisor. Should any harms to participants occur, these will be brought to the attention of the TSC for discussion, and reporting.

### Intervention – group clinical supervision

The intervention in this study is the provision of monthly GCS (by two trained Clinical Supervision facilitators) in the intervention units for up to two years. This is compared to maternity sites with no monthly GCS offered to midwives. Different to supervision of clinical skills, preceptorship, mentoring and coaching, Clinical Supervision is a structured supportive session lasting up to 90 min with a skilled facilitator that allows participants to deeply reflect on their professional lives. The Australian Clinical Supervision Association defines Clinical Supervision as ‘a formal professional relationship between two or more people in designated roles, which facilitates reflective practice, explores ethical issues and develops skills’ [42].

Group Clinical Supervision will be standardised through only two facilitators delivering the intervention throughout the study period (trained in the endorsed Role Development Clinical Supervision model) [43]. Attendance will be optional but strongly encouraged for midwives to reflect on their practice in a safe, confidential and supportive environment. Control sites will have no GCS sessions, but midwives will be recruited to complete the online (or hardcopy) survey tools every six months for the study period.

### Outcome measures

Every six months, the following will be collected: simple demographic information as well as midwifery qualifications, area of primary work, years of service, intention to leave position or profession and number of GCS or other support sessions attended. In addition, workforce data on midwifery staff turnover and sick leave will be collected from the Local Health District workforce departments at each six month period.

*The Australian Midwifery Workplace Culture (AMWoC) instrument* is a 22 item, 6-point Likert scale questionnaire that measures midwives’ perceptions of their workplace culture. Psychometrically tested, it can contribute to understanding the dimensions of a midwifery work environment, including problematic practices and attitudes [37]. Qualitative data will be collected from an open-ended question added to the AMWoC instrument requesting participants to expand on perceptions of their workplace culture.

*The Copenhagen Burnout Inventory (CBI)* is an extensively used 19-item, 5-point Likert scale that measures personal (6 items), work-related (7 items) and client-related levels of burnout (6 items) [38].

*Clinical Supervision Evaluation Questionnaire (CSEQ)* is a validated 14-item questionnaire on a 5-point Likert scale related to three dimensions: the purpose, process and impact of Clinical Supervision [39].

### Sample size

Assuming that 67% of midwives will have moderate or high levels of burnout [18], a sample size of 744 (373 per arm) from twelve maternity sites with an average of 62 midwives per site will achieve 80% power to detect a 15% decrease in moderate or greater burnout with an α level of 0.05 and intra-class correlations of 0.02. Assuming 20% attrition, the target sample size is 894 (447 midwives). This equates to an average of 75 midwives per site.

### Randomisation

Once recruited and ethical clearance gained, the lead CI will perform simple randomisation of the maternity sites to either the intervention or control arm via a computerised method. Those in the intervention arm will receive monthly GCS in their maternity units, and those in the control arm will not. The study design and nature of the intervention means that blinding for staff will not be possible.

### Data collection and management

Six-monthly online survey data collection will occur for all sites using the AMWoC instrument, the CSEQ (for intervention sites), and the CBI; all validated tools (see Outcome Measures above). Hardcopy surveys will be available for participants. Similarly, non-identifiable numerical workforce data on midwifery staff turnover and sick leave will be collected. We will also determine the dose effect – i.e. we will measure how many GCS sessions the midwives have attended (whether study-related or sessions already offered at their workplace), as well as other supportive workplace strategies such as mentoring/coaching.

Data management plans have been lodged within the [*blinded*] Data Management Plan portal. This describes that data is contained within the cloud-based, daily backed-up REDCap platform. Any hardcopy data (hardcopy versions of the online survey) will be transcribed into this platform by the Research Assistant. Similarly, completed consent forms will be scanned and uploaded to REDCap. All data will be transferred and stored for 15 years within the Data Management Plan portal once the project is completed.

Confidentiality will be maintained throughout the management of the survey data. Data will be non-identifiable, coded and de-personalised. All study-related information will be stored securely at the Sponsor site. All participant information will be stored in locked file cabinets in areas with limited access, before being scanned and stored on a cloud-based secure, password protected computer. All administrative forms will be identified by a coded ID number only to maintain participant confidentiality. All records that contain names or other personal identifiers, such as locator forms and informed consent forms, will be stored separately from study records identified by code number. Limited personnel will have access to data (the Chief Investigator, Research assistant and Biostatistician). Project Principal Investigators will have no access to their own site’s data sets.

### Statistical analysis

Data will be analysed using SPSS on an intention-to-treat approach. Initially descriptive statistics will be used to compare the two groups at baseline, recognising that clustered randomisation does not necessarily achieve balance on covariates at the individual level. The primary outcome, the Copenhagen Burnout Inventory (CBI) will be assessed to calculate the percentage of midwives with moderate or higher levels of burnout. This percent will be ascertained for all midwives in both trial arms every six months. The main analysis will be logistic regression using generalised estimating equations (GEE) to test the main hypothesis related to reduction in burnout rate. Secondary analyses will estimate the impact of the programme on the other relevant outcomes. All analyses will be adjusted for the effect of clustering using generalised estimating equations and will also be adjusted for the variable used in stratification. Counts will be analysed with Poisson regression, binary outcomes with logistic regression and continuous scales with linear regression. Although confounding is not expected to be an issue due to the randomisation, any key variables showing large imbalances by randomisation group will be added to the model. Multiple imputation will be performed on missing data to minimise any potential bias. An intention to treat approach will be used.

The qualitative data within the AMWoC survey will be analysed thematically using the six step approach by Braun and Clarke [44].

**Fig. 1:**
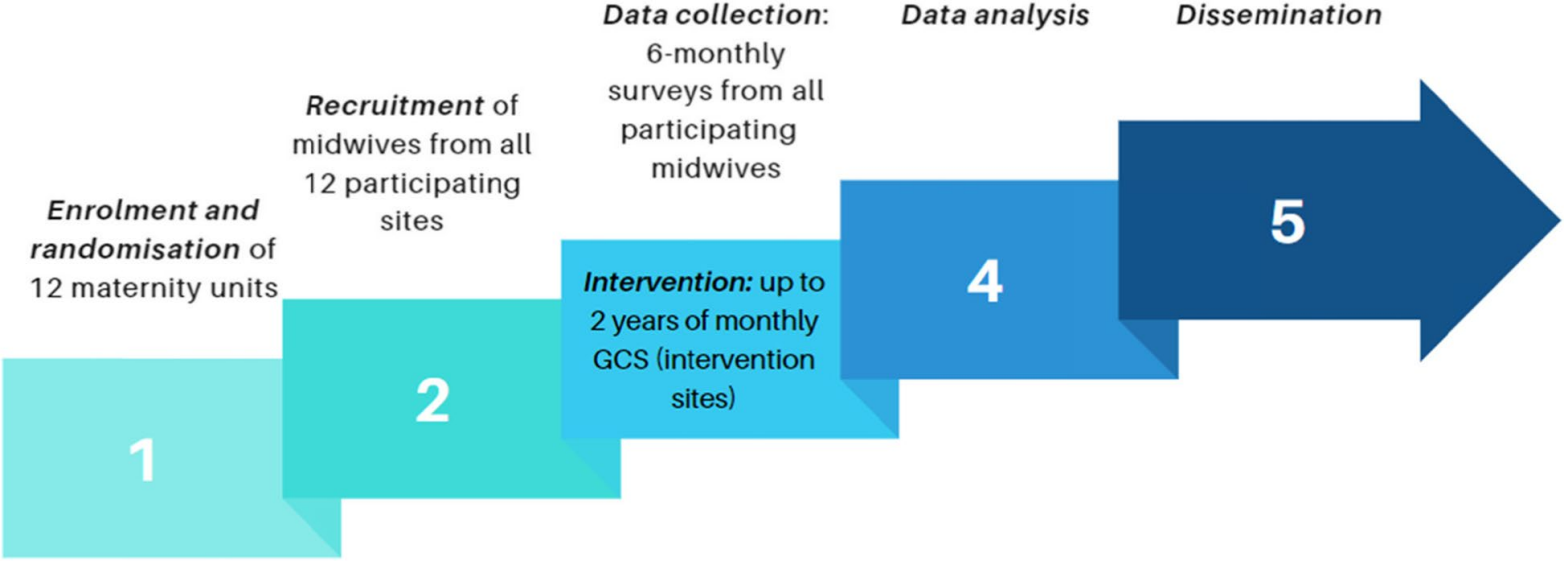
Study progression timeline

Triangulation of quantitative and qualitative data, and the workforce data on turnover and sick leave will provide further insights into the relationship between GCS, midwifery workplace culture and staff turnover. Analyses will occur following each data collection period and amalgamated at the end of the study. The biostatistician responsible for data analysis will be blinded to the maternity site data (within the REDCap database). It is not possible to blind the allocation of the intervention to the maternity site staff and other researchers/Principal Investigators. Figure 1 shows the study progression timeline.

## Discussion

Over 20 years ago it was established that poor care was associated with burnout levels in a large US nursing study [45]. Given midwives are often called obstetric nurses in the US, this includes the midwifery profession. Since then, many studies have found alarmingly high levels of burnout in midwives [11, 18, 21, 46, 47], which has been reported to be higher than burnout in nurses [15]. The current study will be the first cluster RCT to determine whether access and attendance at GCS affects work-related burnout in midwives (the primary outcome). One other quasi-experimental trial did find that Clinical Supervision was beneficial to midwives and obstetricians following only six sessions [48]. Many other observational studies report benefits for midwives [32, 49–53].

Analysis will examine the relationship between perception of the midwifery workplace culture, intentions of midwives to quit their roles/profession, burnout levels and actual staff turnover and sick leave. Again, observational studies have shown the relationship between sick leave and burnout in healthcare workers [15] but this has not been studied with an experimental design before. Sick leave rates can provide an indication of the mental strain/burnout levels of staff, for example, over 50% nurses and midwives take ‘mental health days’ to cope with the demands of their job [54].

The CBI tool has been used extensively to measure burnout in healthcare disciplines. Suleiman-Mantos studied burnout in midwifery, and using the CBI, found 40% had work-related burnout [47]. Similar to the WHELM study [18], this metanalysis showed low client-related burnout, meaning midwives had fewer symptoms of burnout related to caring for women themselves or ‘doing’ midwifery. These studies show that the structure and other system-related aspects of their work are more problematic, causing higher levels of burnout.

## Data Availability

Data from this study will be available to researchers following the conclusion and publication of this study, and after suitable ethical approvals have been undertaken. No later than a year after the conclusion of the study, the Chief Investigator will deliver a completely de-identified data set to the data archival facility for sharing purposes. For data sharing, please contact the lead author of this protocol.

## Abbreviations

GCS: Group Clinical Supervision
CBI: Copenhagen Burnout Inventory
AMWoC: Australian Midwifery Workplace Culture
CSEQ: Clinical Supervision Evaluation Questionnaire

## Footnotes

1 Burnout is characterized by three dimensions: 1) feelings of energy depletion or exhaustion; 2) increased mental distance from one’s job, or feelings of negativism or cynicism related to one’s job; and 3) reduced professional efficacy (WHO, ICD-11 for Mortality and Morbidity Statistics, 2020).

2 Independence is defined as: not part of the same Faculty discipline as any of the applicants or members of the project team; not related to any of the applicants of members of the project team.
